# Re-building the network: mental health system reform in Croatia

**DOI:** 10.3325/cmj.2024.65.65

**Published:** 2024-02

**Authors:** Roberto Mužić, Ana Žegrec, Danijela Štimac Grbić

**Affiliations:** 1 Department for Mental Health and Addiction Prevention, Croatian Institute of Public Health, Zagreb, Croatia; 2 School of Public Health “Andrija Štampar,” University of Zagreb School of Medicine, Zagreb, Croatia

Community-based mental health care is acknowledged as a better alternative to institutionalized care. It improves access to health care services, reduces stigma, better protects human rights, and improves patients’ outcomes (1). The London Declaration highlights the importance of empowering people with mental disorders, advocating that all interventions should be client-oriented, and the focus of care should be on the person's recovery (2).

**Figure 1:**
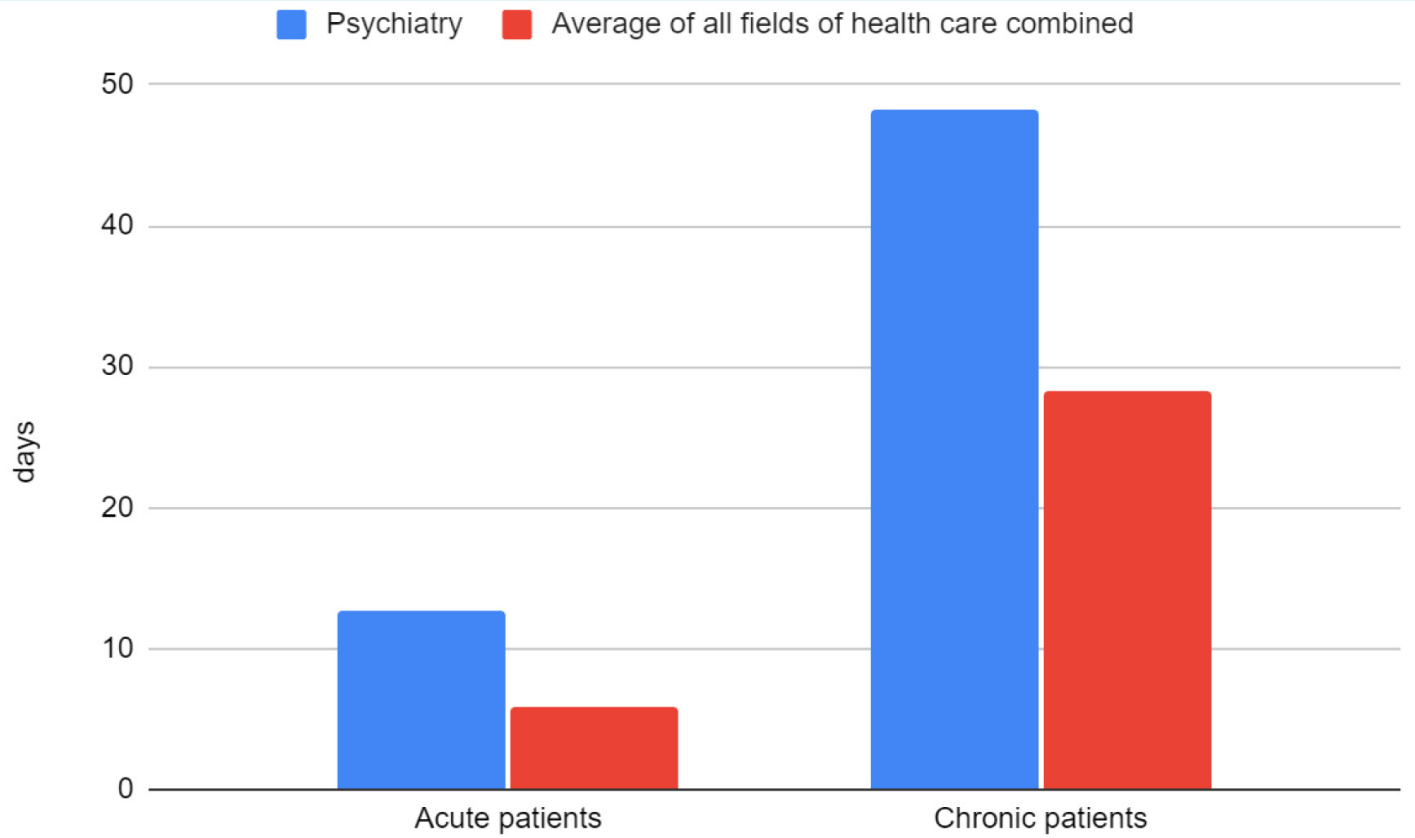
The average length of stay in Croatia.

Yet, some countries, such as Croatia, have a system of psychiatric care that still relies on psychiatric hospitals. Services in the community are limited and are often seen just as post-hospital care. Community-based care should be perceived as a permanent process referring to the period before, during, and after hospital admission, in which the hospital stay is a short intermediate phase, carried out as needed (3). The emphasis should be on recovery as a whole and not just on the regression of symptoms. Literature data support this and indicate a need for change (2). The average length of stay (ALOS) in Croatia for people with mental disorders, both acute and chronic, is among the highest when compared with the ALOS from other fields of care (Figure 1) (4).

**Figure 2:**
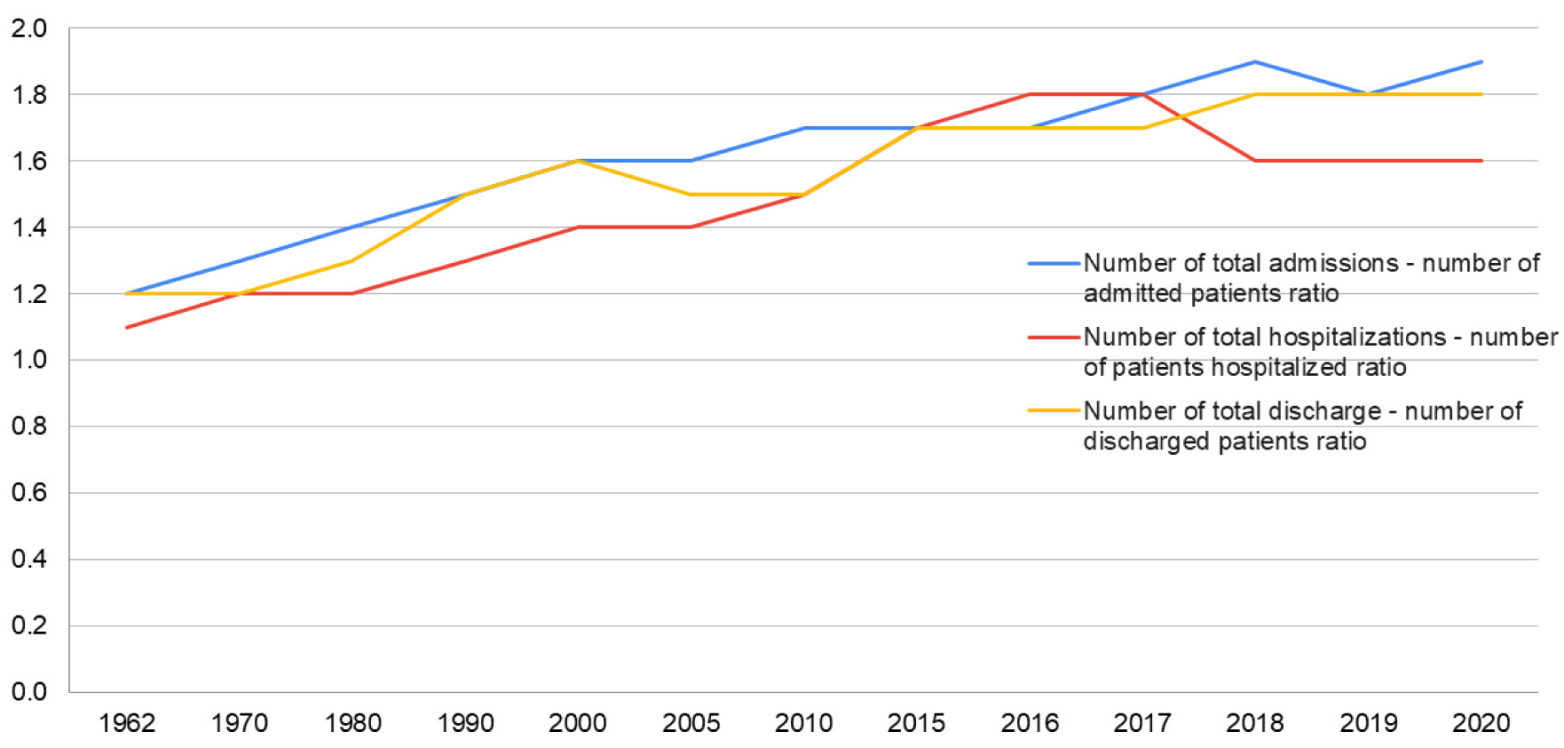
Selected hospital care indicators for persons with schizophrenia or schizoaffective disorders by selected years.

A noticeable trend of “revolving door” hospital admissions is also present. Some patients, especially those with severe mental illness such as schizophrenia, are discharged and admitted in a short period of time, a practice that could increase the hospitalization-patient ratio (Figure 2) (5).

Several interventions toward the establishment of community services have already been made. In 2016 and 2017, the Croatian Ministry of Health and the Trimbos Institute (the Netherlands) conducted a Twinning project called “Ensuring Optimal Health Care for People with Mental Health Disorders.” The project resulted in the creation of the Guidelines for Community Mental Health Care and the Guidelines for Early Recognition and Child and Youth Mental Health (3). The guidelines emphasize an integrated and connected care system, formed by all components and phases of care (3). According to an analysis carried out during the Twinning project, 80% of all patients hospitalized due to mental disorders are admitted through emergency care. This indicates an insufficient recognition of mental disorders and a lack of diagnostic and therapeutic services in primary health care (6). Experience from other countries shows that community-based mental health care services focused on recovery are beneficial for personal and social recovery (7). To improve community mental health care and support, and the transition from the hospital-based to community-based care, the Large-Scale Implementation of Community Based Mental Health Care for People with Severe and Enduring Mental Ill Health in Europe (RECOVER-E) project was conducted in Croatia. The project showed that introducing multidisciplinary community-based mobile teams may increase the availability of mental health services and improve the functional outcomes of service users with severe mental illness (7).

Following RECOVER-E, the Croatian “Strategic Framework for the Development of Mental Health until 2030” was adopted in November 2022. It aims to improve the mental health of the entire population and reorganize the mental health care system from hospital-based to community-based, in accordance with the users’ needs (6). The “Action Plan for the Protection of Mental Health in the Community” was established with an aim of organizing community-based mobile teams and dispensaries for the provision of multidisciplinary care. Accordingly, amendments to the Law on Health Care were adopted, which state that mobile teams can be put in place within the framework of psychiatric services at the primary, secondary, and tertiary levels of health care, and that mental health dispensaries will be established in health centers. The first such dispensaries were established in Split and Zagreb.

In 2021, the “ImpleMENTAL” EU Joint Action (JA-02-2020) started with activities in line with the goals of the Croatian “Strategic Framework.” The reorientation of the mental health care system to a community-based system has already begun by organizing a mobile psychiatric team for interventions in the patient's home led by Vrapče University Psychiatric Hospital.

Several programs and projects have been conducted to promote mental health. Screening programs for mental health risks in primary and secondary schools started in 2022. The “Help To” program increases mental health literacy and educates the staff of primary and secondary schools about interventions in crisis situations. So far, it has been attended by 2000 members of educational staff (6). Partnerships with civil associations such as Croatian Association of Mental Health Associations were established. Experienced experts were involved in the development of new strategic documents, are included in the provision of care as peer workers in the new mobile team, and will be included in other services once they are established. The inclusion of peer workers in service provision has been incorporated in the previously mentioned action plan and law amendments.

For the Croatian network of mental health services to lay the foundations of a balanced model of care, some prerequisites are needed. First, the indication for inpatient hospital admission needs to be properly established to prevent patients with somatic conditions from being treated in psychiatric wards. Second, a sustainable model of supporting accommodation and employment, especially for younger adults with severe mental illness, should be developed (in collaboration with the social care sector, non-governmental organizations, and private sector providers). Third, psychotherapy services must be made more accessible. The Croatian Health Insurance Fund is a sole provider of obligatory health insurance in Croatia. Psychotherapy services are mostly carried out privately and at a higher price than in the public health care system. The Fund should subcontract private psychotherapy service providers to make these services available to the general population. Fourth, a transparent digitized record of indicators of care (for example rates of admissions, both involuntary and voluntary, rates of readmissions) should be reintegrated to help policy-makers better understand the current situation and trends.

In conclusion, although Croatian mental health services are still mostly hospital oriented, some steps toward a balanced model of care have been taken. However, future top priorities should be other interventions toward a strong, integrated, and sustainable system that combines inpatient care with community-based services.
